# Evaluation of Anti-Metastatic Potential of the Combination of Fisetin with Paclitaxel on A549 Non-Small Cell Lung Cancer Cells

**DOI:** 10.3390/ijms19030661

**Published:** 2018-02-27

**Authors:** Anna Klimaszewska-Wiśniewska, Marta Hałas-Wiśniewska, Alina Grzanka, Dariusz Grzanka

**Affiliations:** 1 Department of Clinical Pathomorphology, Faculty of Medicine, Nicolaus Copernicus University in Toruń, Collegium Medicum in Bydgoszcz, 85-094 Bydgoszcz, Poland; d_grzanka@cm.umk.pl; 2 Department of Histology and Embryology, Faculty of Medicine, Nicolaus Copernicus University in Toruń, Collegium Medicum in Bydgoszcz, 85-092 Bydgoszcz, Poland; marciah88@wp.pl (M.H.-W.); agrzanka@cm.umk.pl (A.G.)

**Keywords:** fisetin, paclitaxel, combination therapy, cell migration, cell invasion, non-small cell lung cancer

## Abstract

The identification and development of new agents with a therapeutic potential as well as novel drug combinations are gaining the attention of scientists and clinicians as a plausible approach to improve therapeutic regimens for chemoresistant tumors. We have recently reported that the flavonoid fisetin (FIS), at physiologically attainable concentrations, acts synergistically with clinically achievable doses of paclitaxel (PTX) to produce growth inhibitory and pro-death effects on A549 human non-small cell lung cancer (NSCLC) cells. To further investigate a potential therapeutic efficacy of the combination of fisetin with paclitaxel, we decided to assess its impact on metastatic capability of A549 cells as well as its toxicity toward normal human lung fibroblast. Cell viability, cell migration, and invasion were measured by thiazolyl blue tetrazolium bromide (MTT) assay, wound healing assay, and Transwell chamber assay, respectively. The expression of metastasis-related genes was assessed with quantitative reverse transcriptase real-time polymerase chain reaction (qRT-PCR). Actin and vimentin filaments were examined under the fluorescence microscope. The combination of FIS and PTX significantly reduced cancer cell migration and invasion, at least partially, through a marked rearrangement of actin and vimentin cytoskeleton and the modulation of metastasis-related genes. Most of these effects of the combination treatment were significantly greater than those of individual agents. Paclitaxel alone was even more toxic to normal cells than the combination of this drug with the flavonoid, suggesting that FIS may provide some protection against PTX-mediated cytotoxicity. The combination of FIS and PTX is expected to have a synergistic anticancer efficacy and a significant potential for the treatment of NSCLC, however, further in vitro and in vivo studies are required to confirm this preliminary evidence.

## 1. Introduction

The therapeutic effect of most anticancer drugs relies on their capacity to target cell proliferation and subsequent cell death. Targeting cancer cell migration and invasion has recently received great attention as another approach that holds promise for alternative chemotherapy. However, there are no effective anti-metastatic agents currently available in clinical use [1]. This can be primarily explained by the fact that metastatic cells are difficult to detect, highly aggressive, chemoresistant, and experimentally challenging to model [2]. The ability of cancer cells to disseminate from primary tumor to lymph nodes as well as regional and distant tissues or organs is an inherent feature of malignant tumors that has remained the underlying cause of death in the majority of cancer patients, and, as such, the principal clinical challenge of solid tumor oncology [3]. Considerable progress made during recent years in understanding the intracellular signal transduction pathways that are associated with tumor invasion and metastasis has provided a basis for searching pharmacological agents that effectively block these pathways. It has recently been proposed that one such compound with the potential anti-metastatic activity could be fisetin (3,3′,4′,7-tetrahydroxyflavone; FIS). Fisetin is a naturally occurring member of a flavonol subgroup of flavonoids, commonly found in the human diet especially in fruit like strawberries, apples, grapes, persimmons, and vegetables, such as onions and cucumbers [4].

In several comparative in vitro studies, fisetin has stood out from other dietary flavonoids with its promising and effective antitumor activity against multiple cancer types, with some of the anticancer effects being achieved with low, physiologically relevant concentrations, and without damaging normal cells [5]. The antitumor effect of fisetin has been mainly attributed to its antiproliferative and pro-apoptotic activities [6,7,8,9]. However, there is also accumulating evidence that this flavonoid may exert anti-invasive, anti-migratory and anti-angiogenic effects in vitro. Fisetin-mediated inhibition of metastasis has been shown to occur via suppression of PI3K/AKT and JNK MAPK [10], as well as P38 MAPK-dependent NF-κB signaling pathways [11]. In addition, the modulation of ERK MAPK signaling has also been implicated in fisetin-reduced invasion and the migration of cancer cells [12,13]. This flavonoid was also capable of reversing the molecular changes associated with epithelial-to-mesenchymal transition (EMT) promoted by the Epstein-Barr virus latent membrane protein-1 (LMP1) in CNE1-LMP1 cells [14]. At the cellular level, the anti-migratory [15], as well as anti-angiogenic [16] effects of fisetin, have been related to its microtubule stabilizing abilities. Fisetin has also been shown to potentiate the anti-invasive and anti-metastatic potential of sorafenib in melanoma [17], as well as to synergistically reduce metastatic properties of prostate cancer cells in the combination with cabazitaxel [18].

Paclitaxel (PTX) is one of the most commonly used chemotherapeutic drugs due to its high efficacy against wide varieties of solid tumors and several hematological malignancies. PTX is currently approved by U.S. Food and Drug Administration (FDA) for the treatment of non-small cell lung cancer (NSCLC), breast cancer, ovarian cancer, and AIDS-related Kaposi’s sarcoma. Furthermore, PTX is also often used, either as monotherapy or in the combination with other cytostatic drugs, in patients with thyroid, pancreatic, bladder, head, and neck cancers [19]. Emerging evidence from recent clinical and pre-clinical studies has highlighted the dark side of paclitaxel therapy, namely, its disturbing association with metastasis [20]. Paclitaxel has been shown to promote metastasis in Lewis lung carcinoma (LLC) [21,22], human breast cancer [20,21,23], orthotopic breast tumor [24], epithelial ovarian cancer [25], and leukemia [26] xenograft models as well as syngeneic models of colon and melanoma cancer [27]. The pro-metastatic effects of PTX have been associated with its ability to increase the migration of tumor cells, the number of metastatic nodules, the expression of pro-angiogenic factors SDF-1/CXCL12 and VEGF [22], the amount of invadopodia [28], the number of circulating tumor cells [29], the EMT markers in cancer cells [21,23], the activity of NF-κB [23], the incidence and burden of pulmonary and lymphatic metastasis by TLR4-positive tumors [20], tumor recruitment of pro-metastatic macrophages [30], plasma levels of inflammatory cytokines [31], as well as ALDH+ [22,32] and CD133+ [33] cancer stem cell (CSC) populations. Therefore, although the clinical benefit of paclitaxel in lung cancer patients and others has been clearly shown in many studies, the light shed on its pro-metastatic effects, along with its well-known toxicity, has been encouraging the scientists and clinicians to develop combination therapies that could potentially limit or overcome these drug drawbacks [27,30].

There is growing evidence that dietary polyphenols, due to their abundance in human diet, low cost, a potential lack of toxicity, and a pleiotropic activity, may be the appropriate contenders to serve as a partner for traditional chemotherapeutic drugs [34]. We have recently reported [35] that FIS, at physiologically attainable concentrations, acts synergistically with clinically achievable doses of PTX to produce growth inhibitory and pro-death effects on A549 non-small cell lung cancer cells. The pronounced enhancement of the cytotoxic effect of FIS and PTX had been observed in A549 NSCLC cells, therefore it was of great interest to continue research and verify whether (i) such an effect may be selective for tumor cells and does not affect a normal human lung cell line; (ii) such an effect may be accompanied by suppression of metastatic behavior of A549 cells, or—on the contrary—(iii) such an effect may be associated with potentiation of pro-metastatic potential of these lung cancer cells.

## 2. Results

### 2.1. The Individual and Combined Effect of FIS and PTX on Normal Lung MRC-5 Cells

**Figure 1:**
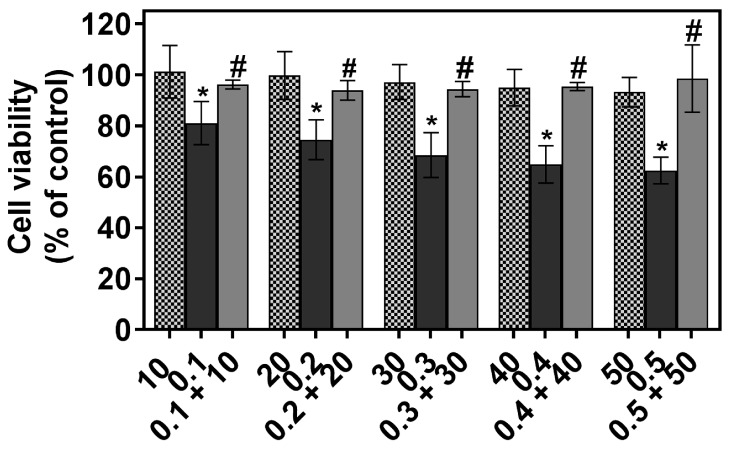
The individual and combined effect of fisetin and paclitaxel on the viability of MRC-5 cells. The cells were treated with various concentrations of paclitaxel (PTX; 0.1–0.5 μM) and fisetin (FIS; 10–50 μM), either alone or in a fixed ratio of 1:100, for 24 h. Control cells (CTRL) were cultured under identical conditions, but without the addition of the tested agents. Cell viability was determined by MTT colorimetric assay. Symbols * and # indicate statistically significant differences compared with control or PTX treatment alone, respectively (*p* < 0.05; One-way ANOVA with Tukey’s post hoc test). All of the values represent the mean ± standard deviation of six independent experiments.

To examine whether the combination of FIS with PTX is toxic for normal human lung cells, MRC-5 fibroblasts were treated with either single or combined agents, and then they were subjected to the MTT assay. As shown in Figure 1, PTX treatment alone was even more toxic to MRC-5 cells than the combination of this drug with FIS, and resulted in a significant dose-dependent decrease in cell viability. Fisetin alone was non-toxic to MRC-5 cells at concentrations up to 50 μM, as it did not affect the survival of these cells (Figure 1). Likewise, the combination of FIS with PTX failed to significantly decrease the viability of normal human lung cells that remained around 95% (Figure 1). These findings suggest that fisetin provides some protection against paclitaxel-mediated toxicity towards MRC-5 normal human lung fibroblasts.

### 2.2. The Individual and Combined Effect of FIS and PTX on the Migration and Invasion of A549 Cells

**Figure 2:**
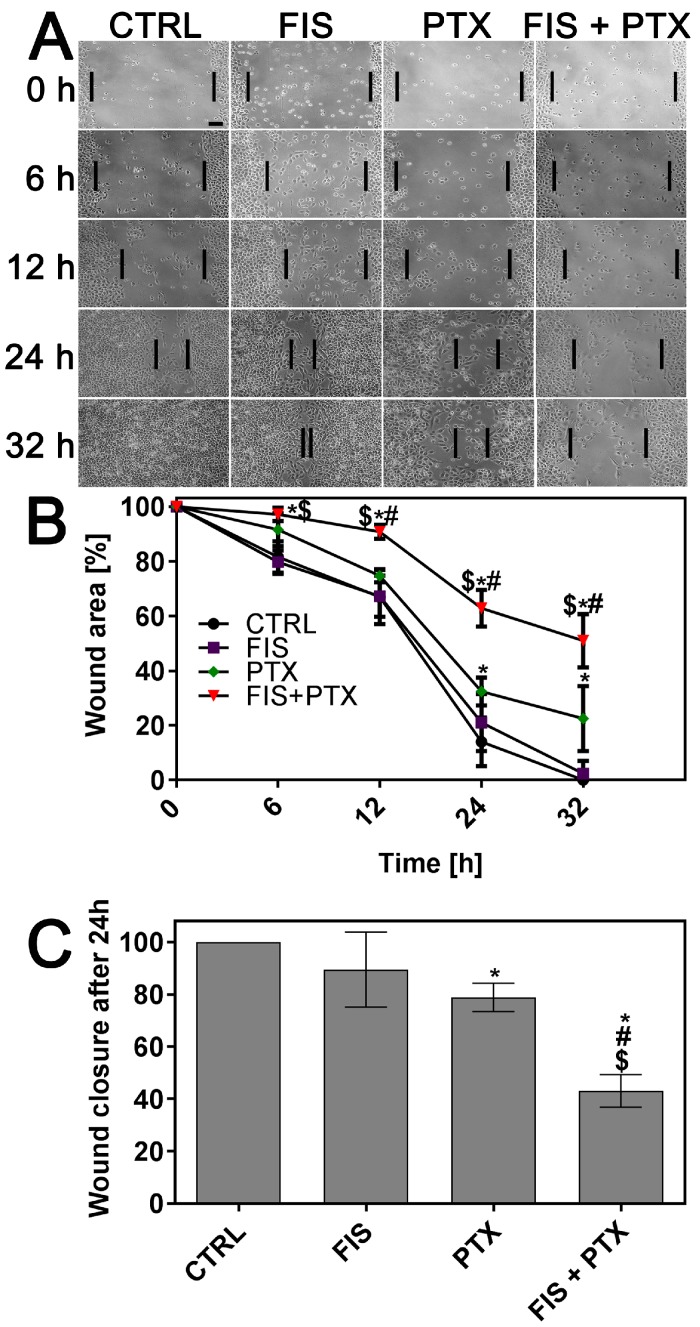
The individual and combined effect of fisetin and paclitaxel on the migration of A549 cells. The cells were treated with 10 μM FIS and/or 0.1 μM PTX and cell migration was assessed by in vitro scratch wound-healing assay. (**A**) Representative images of the scratched areas at different time points were demonstrated, bar = 100 µm; (**B**) The time-course of closure of the wounded areas is shown; and, (**C**) Wound closure at 24 h after treatment as the percentage of control cell migration (set at 100%). Symbols *, $ and # indicate statistically significant differences compared with control, FIS or PTX treatment alone, respectively (*p* < 0.05; One-way ANOVA with Tukey’s post hoc test). Data represent the mean ± standard deviation of three independent experiments with triplicate readings taken from each well.

**Figure 3:**
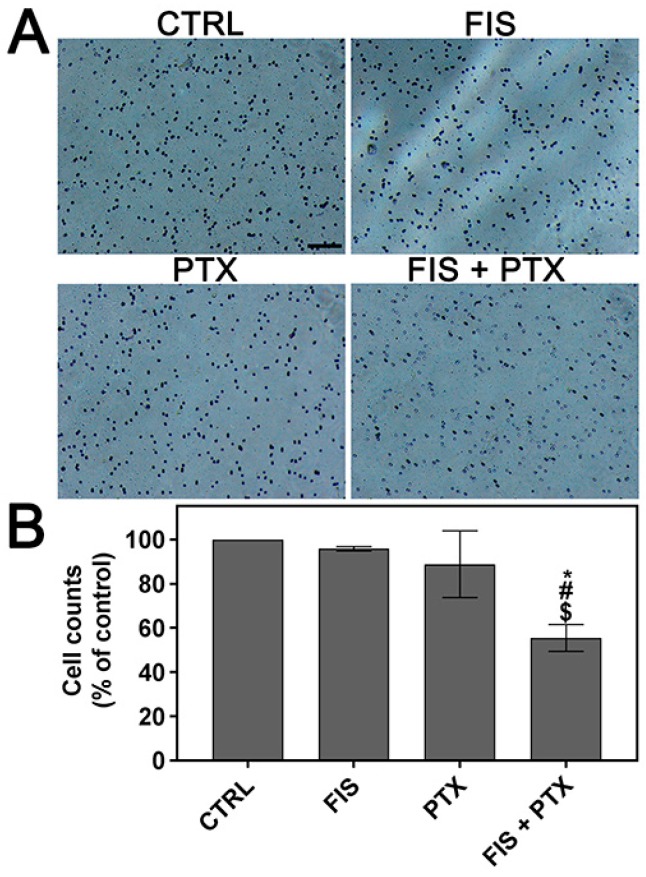
The individual and combined effect of fisetin and paclitaxel on the invasion of A549 cells. The cells were treated for 24 h with 10 μM FIS and/or 0.1 μM PTX and cell invasion was examined by using Matrigel-coated Transwell cell culture chambers. (**A**) Representative images of cells that invaded the underside of the Transwell insert are shown, bar = 100 µm; (**B**) Quantification of invading cells. Symbols *, $ and # indicate statistically significant differences compared with control, FIS or PTX treatment alone, respectively (*p* < 0.05; One-way ANOVA with Tukey’s post hoc test). Data represent the mean ± standard deviation of three independent experiments.

Since cell migration is a crucial step in tumor invasion and metastasis [36], the effect of single and combined treatment on the migratory potential of A549 cells was assessed using in vitro scratch wound healing assay. The process of repopulation of the scratched area by migrating cells was monitored under phase-contrast inverted microscope at regular time intervals (up to 32 h when control cells covered the entire wound surface) and illustrated by representative images in Figure 2A. Whereas, in control cells the wounds were completely repaired after 32 h, detectable (FIS, PTX) or sizeable (FIS + PTX) gaps in the monolayer were still present after the treatment (Figure 2A). The quantitative analysis revealed that the data obtained from A549 cells treated with FIS were statistically insignificant (Figure 2B). Even though PTX was able to significantly reduce the migration capacity of A549 cells, the effect that is produced by its combination with FIS was greater than that of each agent alone (Figure 2B). Figure 2C shows the wound closure at 24 h after treatment as a percentage of control cell migration. At this time point, 89.54 ± 14.33%, 78.89 ± 5.44% and 43.08 ± 6.21% of the wound was filled by the cells treated with FIS, PTX and FIS + PTX, respectively, in comparison to control wound width (Figure 2C). Cell invasion is another essential event in cancer progression and metastasis [37], therefore the effects of FIS and/or PTX and on the invasive ability of A549 cells were evaluated using Matrigel-coated Transwell assay. As shown in Figure 3, the combined treatment resulted in a significant decrease in the number of invaded cells as compared with the effect of either FIS or PTX alone. Based on the number of invaded cells, the flavonoid inhibited invasion of A549 cells by 4.2 ± 0.98%, cytostatic by 11.19 ± 15.12%, and both by 44.55 ± 6.04% as compared to control. The obtained results suggest that when used together, FIS and PTX were more effective in reducing cell migration and invasion than individual agents.

### 2.3. The Individual and Combined Effect of FIS and PTX on Actin and Vimentin Filament Network in A549 Cells

**Figure 4:**
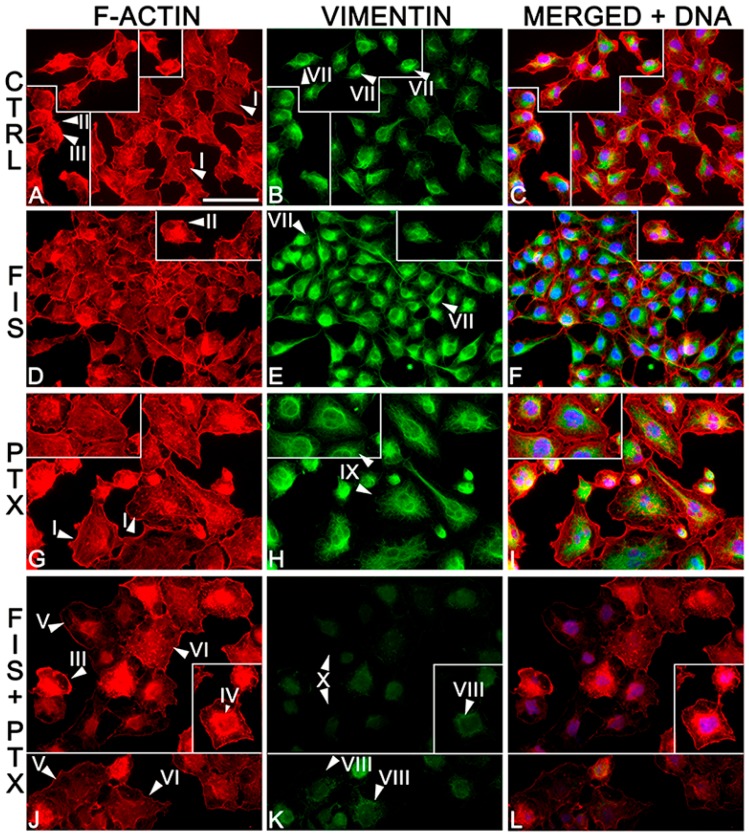
The individual and combined effect of fisetin and paclitaxel on the organization and distribution of actin and vimentin cytoskeleton. The A549 cells were left untreated (**A**–**C**) or treated for 24 h with 10 μM FIS (**D**–**F**), 0.1 μM PTX (**G**–**I**) and the combination of these agents (**J**–**L**). Cytoskeletal proteins as well as cell nuclei were labeled as described in Materials and Methods. Arrowheads indicate (I) stress fibers; (II) filopodia-like protrusions; (III) lamellipodia-like protrusions; (IV) F-actin ring-like structures around the nucleus; (V) disordered actin networks; (VI) the loss of stress fibers; (VII) vimentin rings surrounding the nucleus with clear, large foci located at one region of these structures; (VIII) small dot-like vimentin aggregates located in the perinuclear region; (IX) abundant vimentin filament network; and, (X) a disappearing fluorescent signal for vimentin. The data are representative of two independent experiments. Bar = 50 µm.

Due to the importance of actin and vimentin filaments in cell migration [38,39], it was appropriate to assess the organization and distribution of these cytoskeletal elements in A549 cells treated with FIS and/or PTX. As shown in Figure 4, the tested compounds had a prominent effect on actin cytoskeleton. Control cells were characterized by orderly arranged and well-developed actin filaments, numerous stress fibers, as well as a very thin layer of cortical actin. Moreover, filopodia-like and lamellipodia-like protrusions were abundantly present in these cells (Figure 4A). Fisetin in the combination with PTX resulted in the disorganization of actin cytoskeleton and an almost complete loss of actin stress fibers (Figure 4J). In these cells, F-actin was seen mainly in the form of diffuse staining and only short actin filaments or small punctate accumulations within the cytoplasm (the latter was also observed in control cells). Furthermore, co-treatment with FIS and PTX caused a partial degradation of actin cytoskeleton in the large proportion of A549 cells, and this effect manifested itself as a significant reduction of F-actin fluorescence. There were also few cells with ring-like structures of F-actin accumulated around the nucleus. The filopodia-like and lamellipodia-like protrusions were occasionally observed in co-treated populations, however these structures stained weaklier for F-actin than those seen in control cells (Figure 4J). Such prominent changes were not observed following single treatment with 10 µM FIS, however, an evident decrease in stress fibers could be seen (Figure 4D). There were also an increase in F-actin- and vimentin-rich cell processes that extended over neighboring cells (Figure 4F). The overall F-actin arrangement in cells treated with PTX alone was similar to that of the control cells, although stress fibers in the former cells were thinner than in the latter ones (Figure 4G).

Fisetin and/or PTX had also a marked impact on the structure of the vimentin cytoskeleton. Control A549 cells possessed vimentin mostly in the form of well-organized, dense mesh throughout the cytoplasm, together with thin, often incomplete rings surrounding the nucleus with clear, large foci present at one region of these structures (Figure 4B). The combination treatment led to an almost complete loss of vimentin IF bundles, and this coincided with the formation of small dot-like aggregates located in the perinuclear region, and much more dispersed vimentin network, as well as a very faint and even disappearing fluorescent signal (Figure 4K). Single treatment with fisetin promoted thickening of vimentin rings surrounding the nucleus with enlarged perinuclear foci as compared to similar structures observed in control cells. These alterations were accompanied by a clear increase in the fluorescence intensity in comparison to untreated cells (Figure 4E). Abundant vimentin filament network was also observed following single treatment with paclitaxel. In these cell populations, vimentin formed ring-like structures around the nucleus, which were thicker than those seen in the control cells and often devoid of prominent foci (Figure 4H).

### 2.4. The Individual and Combined Effect of FIS and PTX on the Expression Levels of Metastasis-Related Genes

**Figure 5:**
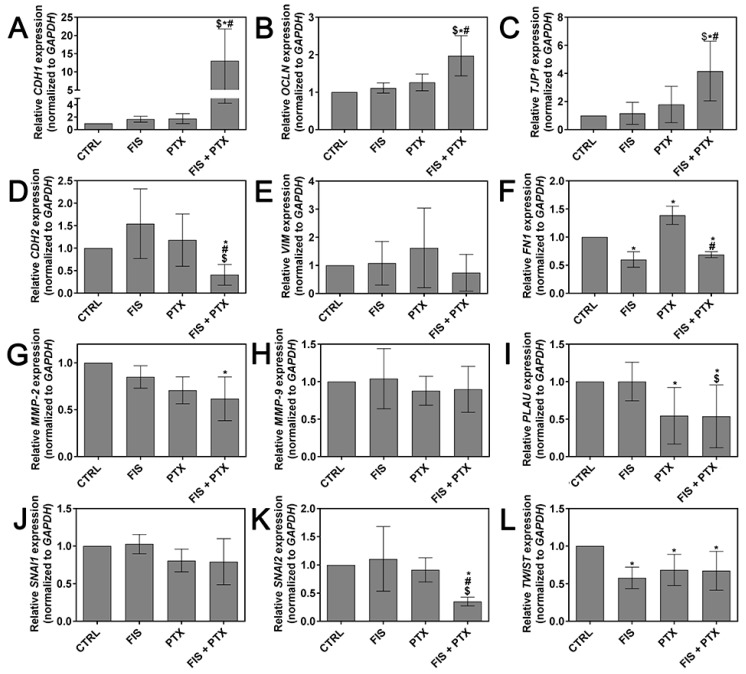
The individual and combined effect of fisetin and paclitaxel on the expression level of metastasis-related genes. The A549 cells were treated for 24 h with 10 μM FIS and/or 0.1 μM PTX. Real-time qRT-PCR measurement of (**A**) E-cadherin, (**B**) occludin, (**C**) ZO-1, (**D**) N-cadherin, (**E**) vimentin, (**F**) fibronectin, (**G**) MMP-2, (**H**) MMP-9, (**I**) uPa, (**J**) SNAIL, (**K**) SLUG, (**L**) TWIST expression level. Relative gene expression was normalized to *GAPDH* housekeeping gene and depicted as a fold difference relative to a calibrator sample (untreated cells; assumed as 1). *, $ and # symbols denote statistically significant differences in comparison with control, FIS or PTX treatment alone, respectively (*p* < 0.05; One-way ANOVA with Tukey’s post hoc test or Kruskal-Wallis with Dunn’s post hoc test). Data represent the mean ± standard deviation of at least three independent experiments.

E-cadherin (encoded by *CDH1* gene), N-cadherin (*CDH2*), fibronectin (*FN1*), matrix metalloproteinase MMP-2/9 (*MMP-2/9*), occludin (*OCLN*), SNAIL (*SNAI1*), SLUG (*SNAI2*), urokinase-type plasminogen activator (uPA, encoded by *PLAU* gene), vimentin (*VIM*), ZO-1 (*TJP1*), and TWIST (*TWIST*) are considered as one of markers of cancer invasion and metastasis, therefore we decided to quantitatively assess the influence of tested agents on their mRNA expression levels. As shown in Figure 5, FIS in the combination with PTX significantly down-regulated the expression of *CDH2*, *FN1*, *MMP-2*, *PLAU, SNAI2*, *TWIST,* and largely up-regulated the transcript level of *CDH1, TJP1,* and less markedly elevated *OCLN* mRNA, but has insignificant inhibitory effect on *MMP-9*, *SNAI1* and *VIM* expression. In the case of *CDH1*, *CDH2, OCLN, SNAI2,* and *TJP1* these modulatory effects were statistically significant not only when compared to control, but also to both individual agents, and for *FN1* only in comparison to cytostatic treatment alone, and for *PLAU* only when compared to single treatment with the flavonoid. Fisetin alone and PTX alone had a differential effect on mRNA expression profile of examined genes, but the vast majority of the changes were not statistically significant. Paclitaxel (0.1 µM) was able to significantly decrease the expression of *PLAU* and *TWIST* mRNA, however it increased the transcript level of *FN1*, while fisetin (10 µM) considerably down-regulated the expression of *FN1* and *TWIST* mRNA (Figure 5). The data indicate that, at concentrations used in the present study, the combination treatment exerted the most pronounced effect on the expression of metastasis-related genes in A549 cells.

### 2.5. The Effect of FIS in the Combination with PTX on the PI3K, AKT and mTOR Expression Level

**Figure 6:**
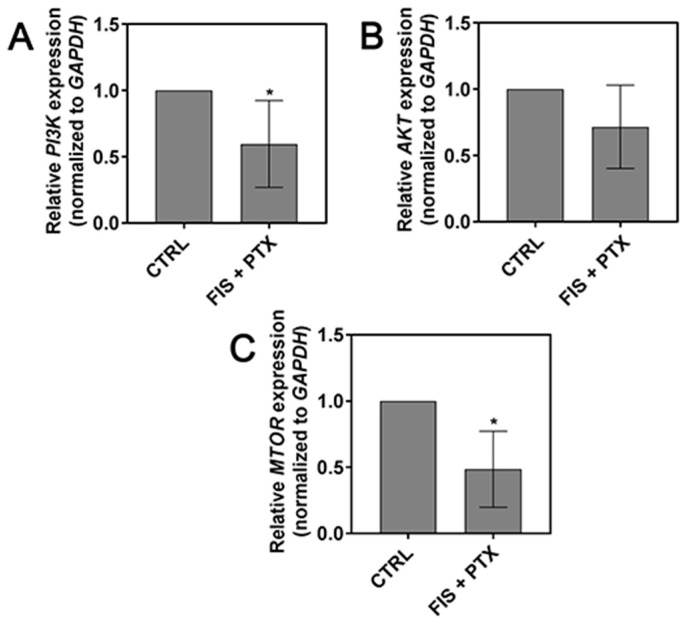
The combined effect of fisetin and paclitaxel on the expression level of PI3K/AKT/mTOR pathway. The A549 cells were co-treated for 24 h with 10 μM FIS and 0.1 μM PTX. Real-time qRT-PCR measurement of (**A**) *PI3K*, (**B**) *AKT*, (**C**) *mTOR* mRNA expression in A549 cells. The expression was normalized to *GAPDH* and presented as a fold difference relative to a calibrator sample (untreated A549 cells; designated as 1). Symbol * indicates statistically significant differences compared with control (*p* < 0.05; one-sample *t-*test). Data represent the mean ± standard deviation of at least three independent experiments.

Given an important role of PI3K/AKT/mTOR signaling pathway in the regulation of tumor cell motility, invasion, and metastasis [40], we asked whether the combination of FIS with PTX (that reduced migration and invasion properties of lung cancer cells) had an impact on the expression of *PI3K*, *AKT*, and *mTOR* mRNA. As depicted in Figure 6, FIS and PTX co-treatment exerted inhibitory effect on mRNA expression of investigated genes, however, in the case of *AKT*, statistical significance was not reached. These results allow us to assume that anti-migratory and anti-invasive effects of FIS and PTX combination may be, at least partially, mediated through the inhibition of PI3K/AKT/mTOR signaling pathway.

## 3. Discussion

To investigate a potential therapeutic efficacy of the combination of the natural flavonoid fisetin with the chemotherapeutic drug paclitaxel, we decided to assess its impact on metastatic capability of A549 non-small cell lung cancer cells, as well as its toxicity toward normal lung fibroblast MRC-5. It was of particular interest to undertake this research since (i) paclitaxel has recently been reported to exert pro-metastatic effect in several in vitro and in vivo cancer models, including lung cancer [20,21,22,23,27,28,31]; (ii) fisetin has been proposed to inhibit the molecular changes associated with EMT induced by LMP1 [14] or the transcription/translation regulatory Y-box binding protein-1 (YB-1) [41]; and, (iii) we have previously shown that FIS plus PTX possessed a potent antiproliferative activity against A549 cancer cells and synergistically reduced viability of these cells through the induction of mitotic catastrophe and autophagic cell death [35]. When it comes to the combination of fisetin with taxanes, Mukhtar et al. have more recently revealed that fisetin and cabazitaxel synergistically reduced the viability and metastatic properties of prostate cancer cells with minimal adverse effects on normal prostate epithelial cells [18].

Emerging evidence has demonstrated that some of chemotherapeutic drugs may induce or accelerate metastasis formation. The focus of most these reports has been on paclitaxel [20,21,22,23,27,28,31], however, there has also been information on other commonly used cytostatics, like cisplatin [27] and cyclophosphamide [42,43,44]. Therefore, there is currently a lot of understanding that anticancer drugs should kill tumor cells as well as prevent metastasis at the same time and a high demand exists for novel mono- and/or combination therapies with an ability to exert such an effect [45,46,47]. Here, we demonstrated that paclitaxel when combined with fisetin significantly reduced cancer cell migration and invasion, at least partially, through a marked rearrangement of actin and vimentin cytoskeleton and the modulation of metastasis-related genes. Noteworthy, most of these effects of the combination treatment were significantly greater than those of individual compounds. More specifically, the combination treatment resulted in a partial disassembly of actin filaments, the complete loss of stress fibers and the overall disorganization of actin cytoskeleton with a concomitant decrease in the fluorescence intensity. Furthermore, F-actin was found to form ring-like structures around the nucleus, suggesting the loss of correct cell polarization, which is a critical step in cell migration [48]. These agents together also led to a dramatic reduction in the density of the vimentin network and its visible rearrangement into perinuclear aggregates. Similar patterns of actin and vimentin rearrangement, accompanied by a decrease of the migratory and invasive behavior of tumor cells have been described in several other studies evaluating anti-metastatic effect of various compounds with potential anticancer activity [2,49,50]. Furthermore, the observed vimentin depletion did not coincide with a decrease in transcript level, which was not significantly changed at the examined time point, suggesting that FIS plus PTX may directly interact with vimentin, leading to its depolymerization/degradation. The combination of FIS and PTX also has only minor effect on *MMP-9* and *SNAI1* mRNA, however it significantly down-regulated the expression of other examined metastasis-promoting markers, such as N-cadherin, fibronectin, MMP-2, uPa, SLUG, TWIST, and markedly up-regulated the mRNA level of metastasis suppressor (epithelial) markers, E-cadherin, occludin, as well as ZO-1. The inhibitory effect of the combination treatment on the migration and invasion of A549 cells could also be assigned to its ability to down-regulate mRNA expression of PI3K, AKT, and mTOR, which are critical signaling proteins implicated in cancer cell growth, proliferation, survival, and metastasis [40]. However, additional studies with a specific inhibitor of PI3K/AKT kinase pathway should be performed to clarify this assumption. It would also be of importance in future research to check the impact of FIS and PTX co-treatment on total and phosphorylated protein levels of PI3K/AKT/mTOR signaling pathway. Moreover, it should be emphasized that the inhibitory effects of FIS + PTX on cell migration and invasion were partly due to its cytotoxic action, given that the viability of A549 cells was also significantly decreased following the combination treatment with 10 µM FIS and 0.1 µM PTX, as we have shown in our previous paper [35]. However, in the present study, we have deliberately chosen these concentrations of tested agents since (i) they are considered as physiologically and clinically achievable, respectively; (ii) they have previously been demonstrated to result in the most potent synergistic anti-tumor effect in A549 cells [35]; (iii) several recent studies have revealed that it was those concentrations of PTX, which inhibited tumor cell growth/induced cell death simultaneously promoted metastasis—in other words—this cytostatic drug may both kill and activate cancer cells, thereby increasing metastasis and chemoresistance [20,27,51]. Therefore, it was important to investigate whether the enhanced antiproliferative effect achieved with the combination of FIS and PTX was not accompanied by an increase in metastatic properties of lung cancer cells.

At the same concentrations as those used in the combinations, fisetin alone (10 µM) and paclitaxel alone (0.1 µM) had a differential effect on examined metastasis-related markers—they inhibited just a few ones, or they allowed to maintain the metastatic phenotype of A549 cells (no influence on tested markers), or—in relation to certain markers—they might even promote a more invasive phenotype. Fisetin neither influenced the migration and invasion of A549 cells nor did it change the transcript levels of most of the examined genes, with the exception of mesenchymal markers *FN1* and *TWIST*, the expression of which was down-regulated. In addition, this flavonoid resulted in no marked alterations in the organization of actin cytoskeleton, however it caused the increase in the density of vimentin filaments with a concomitant increment in the fluorescence intensity of protein staining. Having in mind that vimentin is a canonical marker of EMT, the overexpression of which is correlated with increased tumor growth, a lack of differentiation, invasion, the occurrence of metastases, and a poor prognosis in NSCLC and other tumors [52,53] it seems disturbing that fisetin, at physiologically attainable concentration, resulted in vimentin-rich network in lung cancer cells. Most of the studies devoted to anti-metastatic action of fisetin have displayed that this flavonoid was not able to exert such an effect at the concentrations below 20 µM [11,12,14,15], which is consistent with our results. For example, Li et al. [14] have shown that fisetin suppressed the migration and invasion of LMP1-expressing nasopharyngeal carcinoma cells (CNE1-LMP1) and inhibited molecular changes associated with EMT induced by LMP1. They have demonstrated that while 12.5 µM fisetin had practically no effect on cancer cell migration and invasion, as well as on the expression levels of vimentin and E-cadherin (Western blot/immunofluorescent staining), the effects that are produced by the flavonoid at twice or four times higher concentrations were noticeable and significant [14]. Indeed, we have also found that twice higher concentration of fisetin (20 µM, the dose that is still achievable in vivo [5,54]) led to (i) a significant decrease in the migration of A549 cells; (ii) a partial disassembly of actin filaments and an overall disorganization of actin cytoskeleton with a concomitant decrease in fluorescence intensity; (iii) an almost complete loss of vimentin staining; and, (iv) a significant decrease in mRNA expression of vimentin and N-cadherin, as well as a marked increase in the transcript level of E-cadherin (supplementary Figure S1). Such a distinct effect caused by two different doses of fisetin, both of which are considered physiologically achievable concentrations, make it difficult to predict how effective the flavonoid may be in a clinical setting. The observations of present study, together with our previous findings [35] seem to suggest that the potential therapeutic utility of single-agent fisetin in the treatment of NSCLC is rather limited. However, there are also contrary results. Chien et al. have revealed that very low concentrations of fisetin (≤10 µM) exhibited inhibitory effects on the adhesion, migration, and invasion ability of prostate cancer PC-3 cells, however the impact of 20 µM was more pronounced [10]. In the studies of Liao et al., FIS at concentrations as low as 1–10 µM exerted an inhibitory effect on the adhesion, migration, and invasion of A549 cells through inhibiting the phosphorylation of extracellular signal-regulated kinase 1 and 2 (ERK1/2) and down-regulating the expressions of MMP-2 and uPA at both the protein and mRNA levels [13]. We do not know the reason for the discrepancy between our work and that of Liao et al., but the differences in cell culture conditions (i.e., nutrient levels and the degree of confluence*—*proliferating vs. quiescent cells) might account for it. It is also worth mentioning that a concentration-dependent impact of dietary polyphenols on cancer cell behavior, such as that observed by us, has also recently been reported by others. As an example, Cui et al. have shown that genistein (the dietary soy flavonoid) exerted a dual functional effect on the invasion and metastasis of B16F10 melanoma cells in a dose-dependent manner: higher doses (50–100 µM) of genistein significantly inhibited cell adhesion, migration, and invasion, whereas a lower dose (12.5 μM) had exactly the opposite effect [55].

Similar to 10 µM fisetin, single treatment with paclitaxel had negligible effect on F-actin organization and promoted the development of vimentin filaments in A549 cells. Although PTX was able to significantly decrease the expression of *PLAU* and *TWIST* mRNA, it increased the transcript level of *FN1.* As we mentioned in the Introduction and above, in the Discussion section, paclitaxel has been shown to exert a pro-metastatic effect in a number of in vitro and in vivo cancer models. Based on our results, we cannot unequivocally determine the actual effect of PTX on the metastatic ability of A549 cells, since we used only one concentration of the drug, which, in addition, had no or a differential effect on examined metastasis-related markers. Therefore, we can only assume that although PTX significantly inhibited wound healing, the anti-migratory action of this drug was mainly due to its antiproliferative and cytotoxic effects, rather than the actual impact on cancer cell metastasis process.

Here, we have also demonstrated that fisetin can provide some protection against paclitaxel-mediated toxicity towards MRC-5 normal human lung cells. A similar effect of fisetin, but in relation to nephrotoxicity induced by cisplatin has previously been reported by Sahu et al. [56]. Furthermore, the ability of dietary flavonoids to protect normal cells from the harmful effects of anticancer drugs has been described many times in the literature. For instance, the structural analog of fisetin-quercetin has been shown to provide protection against doxorubicin-mediated toxicity in normal liver L02 cells [57], as well as against melphalan-induced renal and hepatic toxicity in rats [58].

All of the obtained results suggest that the combination of FIS and PTX may have a synergistic anticancer efficacy and a significant potential for the treatment of NSCLC. However, its possible future clinical application is strongly dependent on further in vitro studies on the mechanisms of synergistic action of these agents, and essentially on the in vivo studies, which should be performed to confirm this preliminary evidence.

## 4. Materials and Methods

### 4.1. Cell Culture

The human non-small cell lung cancer cell line A549 (ATCC, Manassas, VA, USA) was maintained in Dulbecco’s Modified Eagle Medium (DMEM; Lonza, Verviers, Belgium). The human fetal lung fibroblast cell line MRC-5 (Sigma-Aldrich, St. Louis, MO, USA) was cultured in Eagle’s Minimum Essential Medium (EMEM; Lonza, Verviers, Belgium). Media were supplemented with 10% fetal bovine serum (PAA, Pasching, Austria), 50 µg/mL gentamycin (Sigma-Aldrich, St. Louis, MO, USA), and 1% non-essential amino acids (only EMEM medium; Sigma-Aldrich, St. Louis, MO, USA). Cultures were carried out in a humidified atmosphere of 95% air and 5% CO_2_ at 37 °C. After reaching about 80% confluence during the exponential growth, the cells were harvested with trypsin–EDTA solution (Sigma-Aldrich, St. Louis, MO, USA) and subcultured on 12- or 6-well plates (BD Falcon, Franklin Lakes, NJ, USA) for further experiments.

### 4.2. Cell Treatment

Stock solutions of FIS and PTX (Sigma-Aldrich, St. Louis, MO, USA) at concentration of 100 mM and 5 mM, respectively, were prepared in 100% dimethyl sulfoxide (Sigma-Aldrich, St. Louis, MO, USA), stored at −25 °C and serially diluted in complete growth medium immediately before use. After a 24-h incubation to allow for cell attachment, 10 µM FIS, and 0.1 µM PTX were added to A549 cells for 24 h as either single or combined agents at a fixed concentration ratio of 1:100. These concentrations of tested agents have previously been shown to result in the highest degree of synergism in A549 cells and therefore they were selected for further drug combination studies on mechanistic aspects [35]. To evaluate the cytotoxic effect of individual and combined treatments on normal human lung cells, MRC-5 fibroblasts were treated with increasing concentrations of FIS (10, 20, 30, 40, 50 µM) and/or PTX (0.1, 0.2, 0.3, 0.4, 0.5 µM) for 24 h.

### 4.3. MTT Assay

The viability of MRC-5 cells was assessed using MTT colorimetric assay, as described previously [35]. Briefly, the cell were seeded in 12-well plates and following a 24-h treatment with FIS and/or PTX and single washing with PBS, the thiazolyl blue tetrazolium bromide (MTT) working solution [prepared by diluting a stock solution of MTT (Sigma-Aldrich, St. Louis, MO, USA; powder dissolved to a concentration of 5 mg/mL in PBS) with DMEM without phenol red (Lonza, Verviers, Belgium) in the ratio 1:9] was added to each well for 3 h (37 °C, 5% CO_2_, 95% air atmosphere). The absorbance was read at 570 nm in a spectrophotometer (Spectra Academy, K-MAC, Daejeon, Korea). Cell viability was calculated as a percentage of MTT reduction relative to untreated cells (designed as 100%). Under standard conditions, MTT assay is used to determine the loss in cell viability resulting from the inhibition of the cell proliferation, the increase in cell death, or the sum of both processes.

### 4.4. Fluorescence of Cytoskeletal Proteins

F-actin and vimentin staining was performed according to previously described protocol [59]. In short, A549 cells grown on sterile glass coverslips were fixed with 4% paraformaldehyde (Serva, Heidelberg, Germany) and permeabilized with 0.25% Triton X-100. F-actin was labeled with phalloidin conjugated to tetramethylrhodamine isothiocyanate (TRITC diluted 1:5 in PBS, 20 min; Sigma-Aldrich, St. Louis, MO, USA). Non-specific background was blocked with 1% bovine serum albumin (BSA). Mouse monoclonal anti-vimentin antibody (diluted 1:50 in BSA-PBS, 60 min; Sigma-Aldrich, St. Louis, MO, USA) was used as a primary antibody to label vimentin (diluted 1:50 in BSA-PBS, 60 min; Sigma-Aldrich, St. Louis, MO, USA), followed by the incubation with a secondary goat anti-mouse antibody conjugated to Alexa Fluor 488 (diluted 1:100 in PBS, 60 min; Sigma-Aldrich, St. Louis, MO, USA). Cell nuclei (DNA) were stained with 4′,6-diamidino-2-phenylindole (DAPI, Sigma-Aldrich, St. Louis, MO, USA). All of the incubations were done at ambient temperature, whereas fluorescent staining was performed in the dark. Finally, slides were mounted in Aqua-Poly/Mount (Polysciences, Warrington, PA, USA) and examined using a Nikon Eclipse E800 fluorescence microscope and NIS-Elements 4.0 software (both from Nikon, Tokyo, Japan).

### 4.5. Scratch Wound-Healing Assay

Cell migration was measured using in vitro scratch wound-healing assay, as described previously [59]. A549 cells were seeded into a 6-well plate and grown to confluence. Confluent cell monolayers were scratched using a sterile 100 μL pipette tip. Images of the same areas were taken immediately after scratching (0 h) and 6, 12, 24, and 32 h later using a DS-5Mc-U1 CCD camera (Nikon, Tokyo, Japan) mounted on a TE100-U inverted microscope (Nikon, Tokyo, Japan). The software used for image acquisition was Nikon NIS*-*Elements (Ver3.30, Nikon, Tokyo, Japan). Cell migration distance was measured by ImageJ software (Ver1.45s, National Institutes of Health, Bethesda, MD, USA).

### 4.6. Cell Invasion Assay

The invasive ability of cells was determined using the Transwell chamber system. The upper side of 24-well polycarbonate filter inserts (8 μm pore size; Corning, NY, USA) was coated overnight with 100 µL of Matrigel (2 mg/mL, diluted in serum-free DMEM; Corning, NY, USA). Treated cells were seeded at a density of 2.5 × 10^5^ in 350 µL of serum-free DMEM medium in the upper compartment of the Transwell insert, and 750 µL of DMEM medium supplemented with 10% FBS was added into the lower chambers as a chemoattractant. All of the cells were incubated for 16 h at 37 °C in a humidified atmosphere with 95% air and 5% CO_2_. After the indicated time, cells on the upper side of the insert were removed with cotton swabs, and those on the underside were fixed with 4% paraformaldehyde, stained with 0.4% crystal violet in 2% ethanol, and photographed using a DS-5Mc-U1 CCD camera (Nikon, Tokyo, Japan) attached to Nikon Eclipse E800 microscope. Each treatment was in triplicate. The invading cell number was quantified by counting at least five random fields.

### 4.7. Quantitative Real-Time PCR Analysis

Total RNA from A549 cells was isolated using Total RNA Mini Plus kit (A&A Biotechnology, Gdynia, Poland), according to the manufacturer’s protocol. The reverse transcription and quantitative PCR reactions were carried out in a single 20-μL LightCycler capillary (Roche Applied Science, Mannheim, Germany) as a one-step real-time qRT-PCR using LightCycler RNA Master SYBR Green I (Roche Applied Science, Mannheim, Germany). The total reaction mixture (20 μL) contained 100 ng of RNA and 0.2 μM of each primer in addition to the LightCycler RNA Master SYBR Green I kit components. The sequences of primers are listed in Table 1. Real-time qRT-PCR (1 cycle of reverse transcription for 20 min at 61 °C, 1 cycle of denaturation for 1 min at 95 °C, and 45 cycles of denaturation for 5 s at 95 °C, followed by annealing and extension for 20 s at 57–60 °C (depending on the melting temperature of the primers) and 5 s at 72 °C, respectively) was run on a LightCycler application system version 2.0 Instrument (Roche Applied Science, Mannheim, Germany). The data obtained from at least three independent experiments were analyzed with LightCycler Software Version 4.0. Relative gene expression was normalized to glyceraldehyde-3-phosphate dehydrogenase (*GAPDH*) internal control and assessed using the ΔΔ*C*t method (2^−ΔΔ*C*t^ method).

### 4.8. Statistical Analysis

All of the values are expressed as mean ± standard deviation, and sta­tistical analysis was carried out with the GraphPad Prism (version 7.01, GraphPad Software, La Jolla, CA, USA). The evaluation of data normality was performed with Shapiro–Wilk test. One-way ANOVA with Tukey’s post hoc comparisons and Kruskal–Wallis test with Dunn’s post hoc comparisons were applied for multiple group comparisons of normally and non-normally distributed data, respectively. For the analysis of differences between two groups, one-sample t*-*test was used when a treated group was compared against an untreated group with a hypothetical value of 1 or 100. Differences were considered statistically significant at *p* < 0.05.

## 5. Conclusions

In summary, the present results demonstrated that the combination of fisetin with paclitaxel inhibited the migration and invasion of A549 human lung cancer cells by modulating the expression of metastasis-related genes and disrupting the structure of actin and vimentin cytoskeleton. In addition, these two agents together were practically non-toxic for human normal lung cells MRC-5. These findings are of particular interest with reference to our previous paper, which showed synergistic antiproliferative and pro-death effects of FIS and PTX on A549 cells. Therefore, when combining the results from both our studies, we may conclude that the combined treatment with fisetin and paclitaxel seems to have apparent benefits over single-drug treatment, as it resulted in the selective enhancement of the cytotoxic effect in lung cancer cells, but not in normal lung fibroblast, and, at the same time, it possessed a potential anti-metastatic activity in these tumor cells. However, our research has left unanswered a number of questions regarding the exact mechanism of the combined action of these agents. Simultaneously, the obtained in vitro results are preliminary and require the confirmation of in vivo significance, if they are to have any clinical utility.

## Figures and Tables

**Table 1:** Details of the investigated and reference genes in the present study.

Genes	Primer Sequences	References
*AKT*	F 5′-GGCTATTGTGAAGGAGGGTTG-3′	[60]
	R 5′-TCCTTGTAGCCAATGAAGGTG-3′	
*CDH1*	F 5′-GCCGAGAGCTACACGTTCAC-3′	[61]
	R 5′-ACTTTGAATCGGGTGTCGAG-3′	
*CDH2*	F 5′-GCTTCTGGTGAAATCGCATTA-3′	[62]
	R 5′-AGTCTCTCTTCTGCCTTTGTAG-3′	
*FN1*	F 5′-ACTGCCCACTCCTACAACCA-3′	[63]
	R 5′-TCTGCGAACACCACTCCA-3′	
*GAPDH*	F 5′-ACAACTTTGGTATCGTGGAAGG-3′	[64]
	R 5′-GCCATCACGCCACAGTTTC-3′	
*MMP-2*	F 5′-GATACCCCTTTGACGGTAAGGA-3′	[65]
	R 5′-CCTTCTCCCAAGGTCCATAGC-3′	
*MMP-9*	F 5′-CCCTGGAGACCTGAGAACCA-3′	[66]
	R 5′-CCCGAGTGTAACCATAGCGG-3′	
*OCLN*	F 5′-CCCCATCTGACTATGTGGAAAGA-3′	[67]
	R 5′-AAAACCGCTTGTCATTCACTTTG-3′	
*PI3K*	F 5′-AGTAGGCAACCGTGAAGAAAAG-3′	[68]
	R 5′-GAGGTGAATTGAGGTCCCTAAGA-3′	
*PLAU*	F 5′-GGGAATGGTCACTTTTACCGAG-3′	[69]
	R 5′-GGGCATGGTACGTTTGCTG-3′	
*SNAI1*	F 5′-TTCAACTGCAAATACTGCAACAAG-3′	[70]
	R 5′-CGTGTGGCTTCGGATGTG-3′	
*SNAI2*	F 5′-TGTGACAAGGAATATGTGAGCC-3′	[71]
	R 5′-TGAGCCCTCAGATTTGACCTG-3′	
*MTOR*	F 5′-TCACATTACCCCCTTCACCA-3′	[72]
	R 5′-TCAGCGAGTTCTTGCTATTCC-3′	
*TJP1*	F 5′-ACCAGTAAGTCGTCCTGATCC-3′	[64]
	R 5′-TCGGCCAAATCTTCTCACTCC-3′	
*TWIST*	F 5′-GTCCGCAGTCTTACGAGGAG-3′	[73]
	R 5′-GCTTGAGGGTCTGAATCTTGCT-3′	
*VIM*	F 5′-GTTTCCAAGCCTGACCTCAC-3′	[74]
	R 5′-GCTTCAACGGCAAAGTTCTC-3′	

## Supplementary Materials

Supplementary materials can be found at http://www.mdpi.com/1422-0067/19/3/661/s1.

## Abbreviations

FIS	fisetin
PTX	paclitaxel
NSCLC	non-small cell lung cancer
EMT	epithelial-to-mesenchymal transition
