# Supplementary material for: Evaluation of Anti-Metastatic Potential of the Combination of Fisetin with Paclitaxel on A549 Non-Small Cell Lung Cancer Cells
Source: Int J Mol Sci. 2018 Feb 27;19(3):661. doi: 10.3390/ijms19030661 (PMC5877522; doi:10.3390/ijms19030661)
Supplement: Supplementary file 1 [file ijms-19-00661-s001.pdf]

Supplementary Materials: Evaluation of Anti-Metastatic Potential of the Combination of Fisetin with Paclitaxel on A549 Non-Small Cell Lung Cancer Cells

Anna Klimaszewska-Wiśniewska, Marta Hałas-Wiśniewska, Alina Grzanka and Dariusz Grzanka

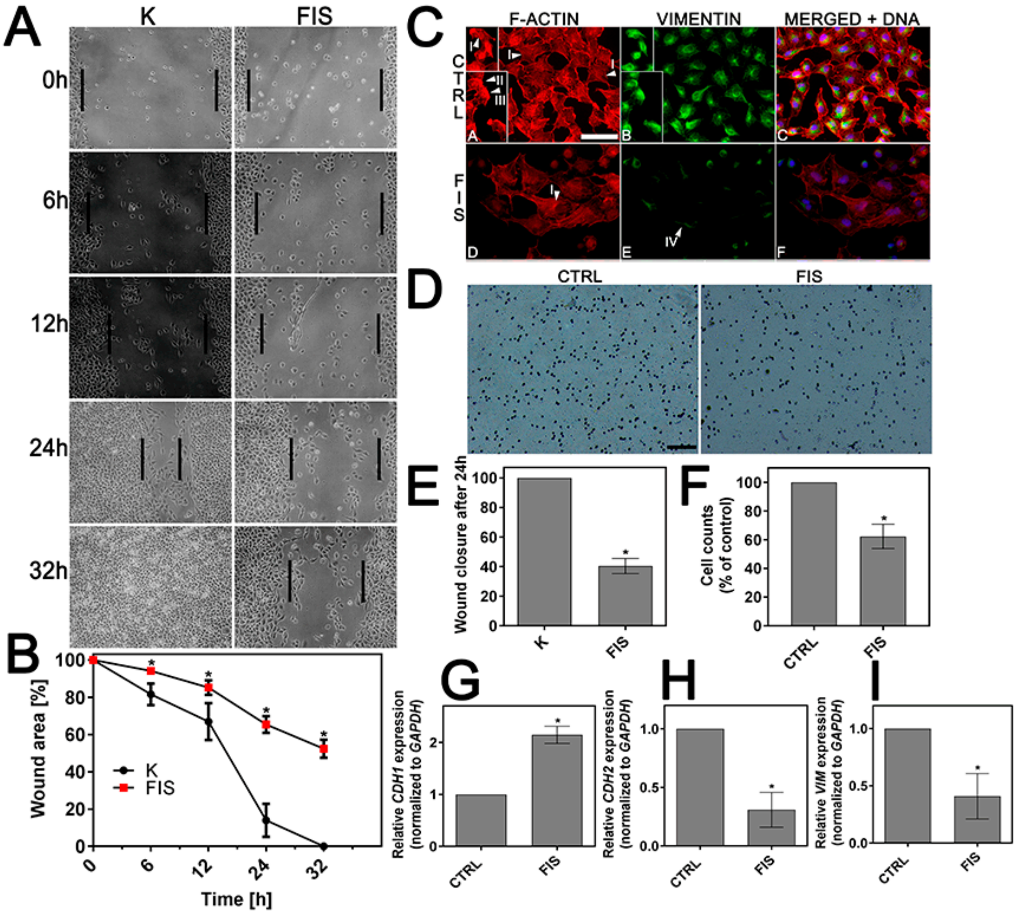

Figure S1. The effect of fisetin on the metastatic potential of A549 cells. The cells were treated for 24 h with 20 μM fisetin (FIS) or left untreated (CTRL). (A–B,**E**): Cell migration was examined by in vitro scratch wound-healing assay. (**A**) Representative images of the scratched areas at different time points were demonstrated; (**B**) The time-course of closure of the wounded areas is shown; E Wound closure at 24 h after treatment as the percentage of control cell migration (set at 100%). (**C**) The organization and distribution of actin and vimentin cytoskeleton was examined as described in Materials and Methods. Arrowheads indicate (I) stress fibers; (II) filopodia-like protrusions; (III) lamellipodia-like protrusions; (IV) a disappearing fluorescent signal for vimentin. Bar = 50 μm. (**D**,**F**): Cell invasion was examined by using Matrigel-coated Transwell cell culture chambers. (**D**) Representative images of cells that invaded the underside of the Transwell insert are shown. Bar = 100 μm; (**F**) Quantification of invading cells. G-I: Real-time qRT-PCR measurement of G E-cadherin, H N-cadherin, I vimentin mRNA expression in A549 cells. The expression was normalized to GAPDH and presented as a fold difference relative to a calibrator sample (untreated A549 cells; designated as 1). Symbol * indicates statistically significant differences compared with control ($p < 0.05$; (**B**) One-way ANOVA with Tukey's post hoc test or (**E**–**I**) one-sample *t*-test). Data represent the mean ± standard deviation of at least three independent experiments.
